# Validation Study on Risk-Reduction Activities after Exposure to a Personalized Breast Cancer Risk-Assessment Education Tool in High-Risk Women in the WISDOM Study

**DOI:** 10.21203/rs.3.rs-2787493/v1

**Published:** 2023-05-10

**Authors:** Tianyi Wang, Mandy Che, Yash S Huilgol, Holly Keane, Deborah Goodman, Rashna Soonavala, Elissa Ozanne, Yiwey Shieh, Jeffrey K Belkora, Allison Stover Fiscalini, Athena Breast Health Network Investigators and Advocate Partners, Laura J Esserman

**Affiliations:** 1. UC San Francisco Department of Surgery, San Francisco, USA; 2. University of Michigan Medical School, Ann Arbor, USA; 3. Rush University Medical College, Chicago, USA; 4. UC San Francisco School of Medicine, San Francisco, USA; 5. Peter MacCallum Cancer Centre, Melbourne, Australia; 6. UC Irvine Department of Epidemiology, Irvine, USA; 7. University of Utah School of Medicine Department of Population Health Sciences, Salt Lake City, USA; 8. Weill Cornell Medicine Department of Population Health Sciences, New York, NY, USA

**Keywords:** personalized, risk reduction, breast cancer, genetic counseling, education aid, decision making, validation study

## Abstract

We performed a 318-participant validation study of an individualized risk assessment tool in women identified as having high- or highest-risk of breast cancer in the personalized arm of the Women Informed to Screen Depending on Measures of risk (WISDOM) trial. Per protocol, these women were educated about their risk and risk reducing options using the Breast Health Decisions (BHD) tool, which uses patient-friendly visuals and 8th grade reading level language to convey risk and prevention options. Prior to exposure to the educational tool, 4.7% of women were already taking endocrine risk reduction, 38.7% were reducing alcohol intake, and 62.6% were exercising. Three months after initial use of BHD, 8.4% of women who considered endocrine risk reduction, 33% of women who considered alcohol reduction, and 46% of women who considered exercise pursued the risk-reducing activities. Unlike lifestyle interventions which are under the control of the patient, additional barriers at the level of the healthcare provider may be impeding the targeted use of endocrine risk reduction medications in women with elevated breast cancer risk.

## INTRODUCTION

Breast cancer is the second leading cause of cancer death in the United States and the most common cancer in women, with one in eight (12.3%) women developing breast cancer in their lifetime.^1^ While there are effective strategies for breast cancer prevention with level 1 evidence, there is little evidence that the women who would stand to benefit most are being counseled. Current strategies to identify women at higher risk include genetic testing of women with strong family histories, and recommendations for more intensive surveillance or prophylactic surgery in women found to be mutation carriers. The vast majority of women are not mutation carriers, but many still have risk and are not routinely screened. For women found to be at elevated risk, there are several strategies to reduce risk, including lifestyle interventions (reduction of alcohol intake, increasing exercise, weight loss), the use of endocrine risk reduction medications (selective estrogen receptor modulators and aromatase inhibitors), and avoidance of combined hormone replacement after menopause.^1–16^ While lifestyle modifications are recommended for all women, randomized controlled clinical trials support the addition of endocrine risk reduction in women at high risk of developing breast cancer.^2,17–20^ The United States Preventative Task Force guidelines encourage primary care providers to identify high risk women and offer endocrine risk reduction.^18^ Risk models including Gail used in the Breast Cancer Risk Assessment Tool, Tyrer-Cuzick, BOADICEA, and Breast Cancer Surveillance Consortium (BCSC) help to stratify breast cancer risk using factors such as age, reproductive history, prior disease, family history, and breast density.^21–28^ Despite clinical guidelines, availability of risk models, and multiple FDA approved endocrine risk reducing medications, uptake of breast cancer endocrine risk reduction in the United States remains low.^20^

Only a small portion of women eligible for risk reducing medications receive treatment due to lack of education, low health literacy, concerns about side effects, aversion to medication, cost, and misconceptions about risks and benefits of treatment.^5,20,29–31^ Educational risk assessment tools allow people to understand their personal risk and weigh the risks and benefits of risk reducing activities.^32^ In the clinical setting, educational tools can facilitate individualized shared decision-making approaches with providers to improve risk reducing medication uptake in women who would benefit.^18^

Previously, Keane and Huilgol et al. described the creation and pilot study of the Women Informed to Screen Depending on Measures of risk (WISDOM) Risk Assessment Tool that educates high- and highest-risk women on their personal breast cancer risk and risk-reducing strategies using personalized genetic testing results, patient-friendly visuals, and 8th grade reading level language.^33,34^ The purpose of developing the tool was to deploy a risk assessment tool to aid women in considering and pursuing risk-reducing activities, and to learn if high risk women would be particularly compelled to pursue endocrine risk reduction. The broader aim was to assess whether the risk-assessment tool would ease anxiety about breast cancer risk by providing actionable risk reduction steps and to determine if understanding risk would reduce breast cancer anxiety in the high and highest-risk groups. While the pilot study evaluated high- and highest-risk women’s”immediate desires to pursue risk-reducing activities after using the tool, it did not determine whether they truly implemented the strategies.

Here, we describe results of the validation study of the WISDOM Study risk assessment tool in women of high and highest breast cancer risk. The study builds upon our previous pilot study by not only comparing efficacy of a new educational risk assessment tool between high and highest breast cancer risk groups but also temporally evaluating uptake of risk reducing strategies through an immediate feedback and three-month follow up survey. Through this unique lens, we hope to further our understanding of the following questions:

1. Is the use of the WISDOM Study risk assessment tool in high- and highest-risk women associated with changes in health-related behavior and uptake of endocrine risk reduction?
2. What are barriers to health-related behavior change and endocrine risk reduction uptake among high- and highest-risk women following use of an educational risk assessment tool?
3. To what extent does an educational risk-assessment tool affect breast cancer anxiety in high and highest breast cancer risk women?

## RESULTS

### Risk Assessment Tool Validation Study Participants

The validation study included 318 WISDOM study participants who were classified as elevated risk in the top 2.5% of BCSC score by age group, which corresponds to high-risk women recommended annual screening or highest-risk women recommended every six-month screening (Table 1). Average BCSC scores for high- and highest-risk women in the study are 5.10 and 7.62 respectively. 109 of the 318 participants responded to the three-month follow up survey. Participants were predominantly white, college graduate or higher, between ages 50 – 69, with BMI 18.5 – 24.9 (Table 1).

### Risk Reduction Activities After Use of Breast Health Risk Assessment Tool

The majority of participants (98.4%) believed that the tool helped them understand their breast cancer risk (Supplementary Table 1). To evaluate risk-reduction activities, we assessed patient reported risk-reducing activity (endocrine risk reduction, alcohol reduction, and exercise) across three time points: before using tool, considerations immediately after using tool, and activities that were implemented 3 months later. Before using the tool, 4.7% of women were taking endocrine risk reduction, 38.7% were reducing alcohol intake, and 62.6% were exercising (Table 2). Immediately after using the tool, 34.6% of women surveyed considered endocrine risk reduction, 14.8% considered decreasing alcohol use and 30.8% considered increasing exercise (Table 2). Next, we examined whether a greater proportion of individuals who considered a risk-reducing activity after using the decision tool pursued it three months later compared to those who did not initially consider it (Supplementary Tables 3a - c). For endocrine risk reduction, 4 out of 48 women (8.4%) who considered it began taking endocrine risk reduction three months later, while 8 out of 61 (13.1%) who did not consider it began taking endocrine risk reduction three months later (Supplementary Table 3a). For alcohol reduction, 31 out of 93 women (33.3%) who considered reducing began to do so three months later, while 11 out of 16 (68.7%) who did not consider it began three months later (Supplementary Table 3b). Lastly, 39 out of 85 women (45.9%) who considered exercising more did so three months later while 14 out of 24 women (58.3%) who did not consider it began three months later (Supplementary Table 3c).

Three months after women first used the Breast Health Decisions tool, we asked them whether they discussed their risk with their provider and what risk-reducing activities their provider recommended. A total of 80 (73.3%) women out of the 109 who submitted a three-month follow up survey discussed their breast cancer risk with their provider (Table 3). Healthcare providers recommended endocrine risk reduction to 17% of high- and highest-risk women, alcohol reduction to 14%, and increased exercise to 20% (Table 3). These recommendation percentages were not significantly different between high- and highest-risk women (Table 3).

### Barriers to discussing risk with provider and using risk-reducing strategies

The most common reason for not discussing one’s risk with a provider was the “other” category, with most participants stating that they have not had their appointment or risk reduction was not brought up during their appointment (Supplementary Table 4). The most commonly selected barriers to endocrine risk reduction were “other” and “fear of side effects” (Supplementary Table 4). Within the “other” category, most women stated that the provider did not recommend the medication. Furthermore, a majority of women who were not reducing alcohol intake or increasing exercise were not doing so because they were already performing the risk-reducing activities (Supplementary Table 4).

### Emotional Well Being after use of Risk Assessment Tool

The breast health risk assessment tool eased anxiety about breast cancer risk in 43.7% of participants. A similar proportion of women (38.4%) felt neutral about the tool’s impact on their anxiety. Women who thought the tool did not ease their anxiety made up 16.3% of the surveyed participants (Supplementary Table 1). After stratifying for breast cancer risk, no difference between high and highest-risk women were found (Fig. 4).

When asked about the frequency women worried about their breast cancer risk three months after first using the decision tool, 5.5% often worried, 48.6% of women sometimes worried, and 45% did not worry at all (Supplementary Table 2). After stratification for breast cancer risk level, no difference between high- and highest-risk women were found (Fig. 5).

## DISCUSSION

### Similar risk reduction strategies across risk groups and persistent downstream barriers to endocrine risk reduction

**Figure 1:**
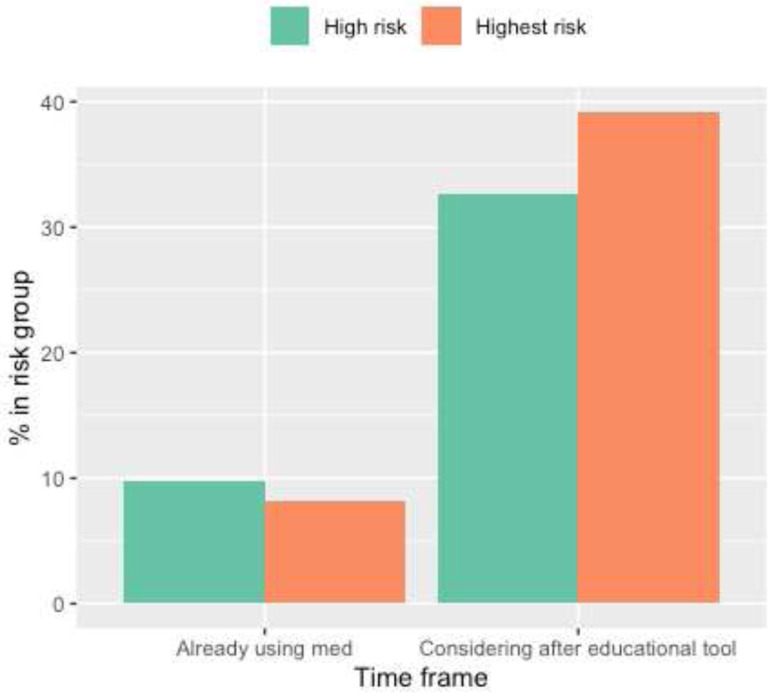
Endocrine Risk Reduction Use and Considerations Bar graph of endocrine risk reduction use and considerations of reducing alcohol in high and highest breast cancer risk participants. Data collected from immediate feedback survey. Note: N/A

**Figure 2:**
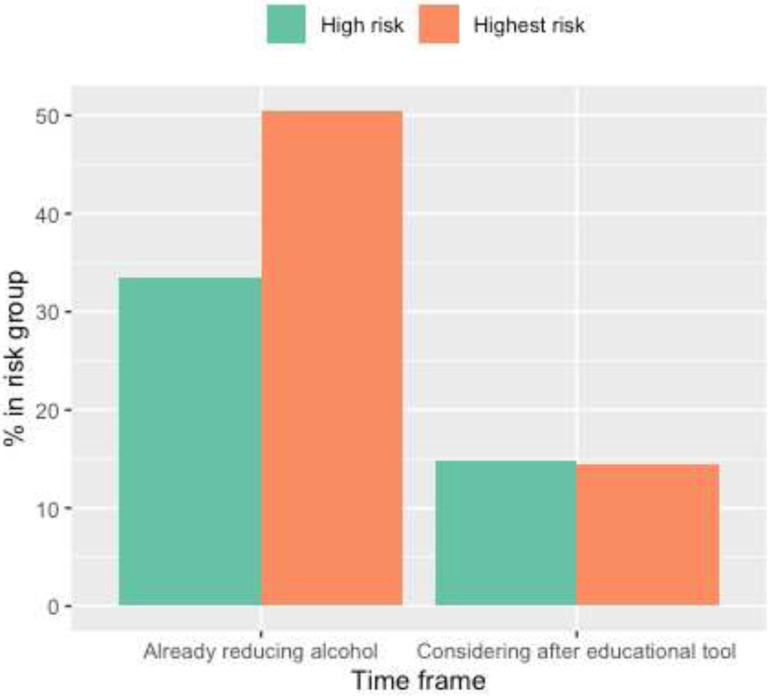
Alcohol Reduction Use and Considerations Bar graph of alcohol reduction and considerations of reducing alcohol in high and highest breast cancer risk participants. Data collected from immediate feedback survey. Note: N/A

**Figure 3:**
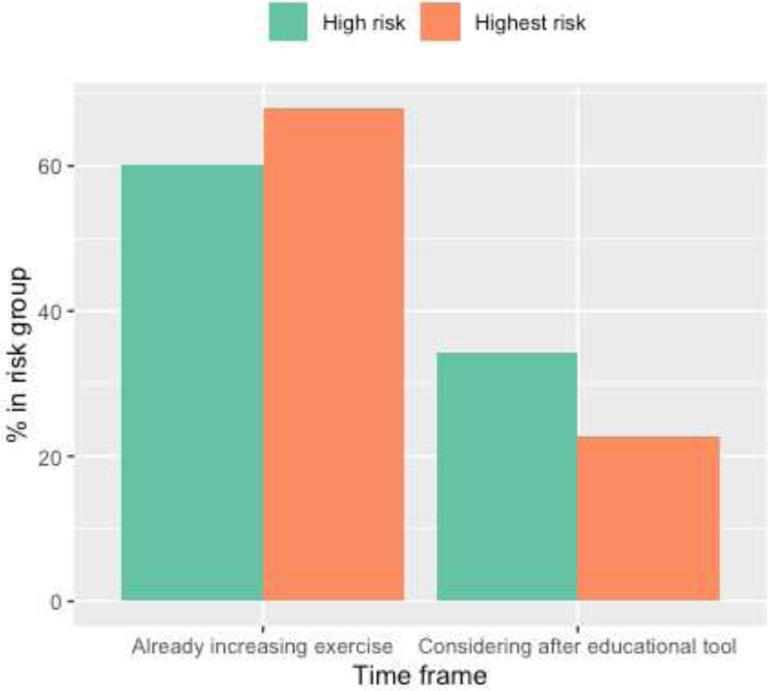
Exercise Use and Considerations Bar graph of exercise use and considerations of pursuing exercise in high and highest breast cancer risk participants. Data collected from immediate feedback survey. Note: N/A

While our initial results are promising, our data also suggests that factors other than initial risk assessment education continue to influence final risk-reduction decisions. To illustrate, a large proportion of all participants (30–40%) considered endocrine risk reduction after using the tool, however the proportion of women taking endocrine risk reduction three months later remains significantly less than those who considered the medication (Fig. 1–3, Table 2). In fact, only 8.4% of women who considered endocrine risk reduction pursued it three months later compared to 30–50% of individuals who considered lifestyle modification (Supplementary Tables 3a-c). Furthermore, the use of endocrine risk reduction was not statistically different between high- and highest-risk women (Fig. 1–3).

Lifestyle interventions are under control of the patient while endocrine risk reducing strategies require the support and intervention of a primary care physician or breast cancer prevention specialist. The majority of women who did not pursue endocrine risk reducing medication reported that they either did not have a follow up visit with their primary care physician, or the topic was not brought up. These results suggest that women continue to face barriers to pursue endocrine risk reduction despite becoming more educated and having a desire to take the medication after using the risk assessment tool. There was no active outreach to the participants’” physicians regarding the results of the risk assessment and BHD tool, thus it is also unclear how many of the participants were considered to have elevated risk by their primary care physician. To that end, highest risk women do not have higher uptake of endocrine risk reduction than high risk women after using the educational risk assessment tool.

We did not capture all of the barriers to medication use after the session using the risk assessment tool. Prior papers have suggested that there are barriers to endocrine risk reduction uptake at the provider level in the clinic.^29,30,35^ Past literature indicates that when assessing risk, most providers never calculate Gail scores (76%).^35^ While many providers discuss increased risk to high risk women (58%) and tailor screening based on risk (53%), fewer providers usually or always discuss endocrine risk reduction (13%).^35^ Challenges faced by providers include lack of confidence in risk assessment and knowledge, identifying suitable candidates for preventative strategies, insufficient knowledge of risk-reducing medications, more immediate issues, and lack of time during clinic visits.^29,30,35^ Despite our efforts in providing a printout summarizing their risk for women to bring to their appointments, this information does not appear to be routinely shared with the primary care physicians. Even when identified as high risk by our study, women are still not getting counseling at the level of their primary care physician, which further confirm the existing literature that indicates that providers are not consistently assessing risk, discussing it, and recommending endocrine risk reduction to high- and highest-risk women who could benefit. Therefore, despite clinical guidelines, providers may not be targeting high-risk women interested in endocrine risk reduction for discussions. Furthermore, when asked about barriers to taking medication, many women noted that their provider did not recommend doing so and that they listen to what their provider recommends (Supplementary Table 4). Since primary care providers are often women’s most trusted source of health information, application of breast cancer risk assessment tools in the clinical setting will require education of and collaboration with the healthcare providers directly involved in patient care.^31,36,37^ This proposal would emulate the adoption of heart disease risk assessment by primary care physicians, who then implemented interventions to reduce risk for heart attack and stroke, resulting in reducing the risk of cardiac related mortality by 50% over the past several decades.^38,39^ Alternatively, providing women with virtual prevention clinics could improve medication uptake.

### Emotional Well Being after use of Tool Depends on Risk Group

**Figure 4:**
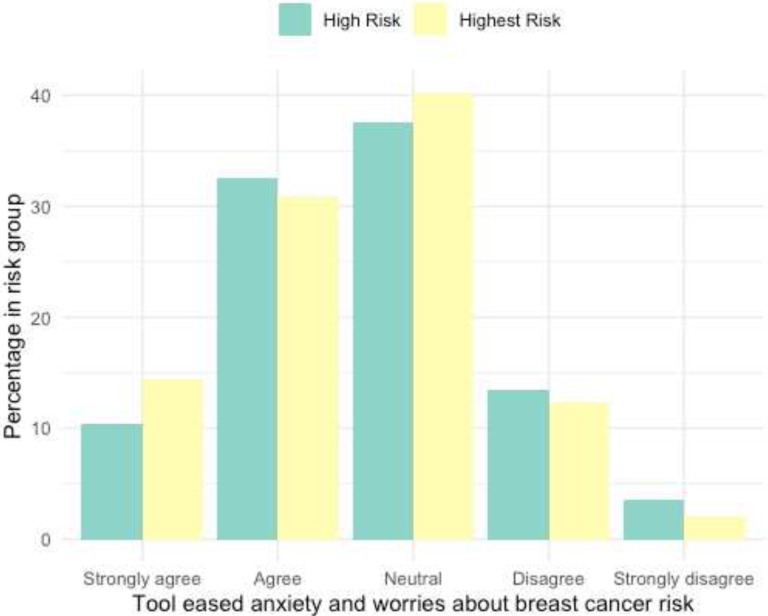
Risk-Assessment Tool and Anxiety about Breast Cancer Risk (immediately after use) Bar graph of anxiety and worry about breast cancer risk after use of tool (from feedback survey). Responses obtained through Likert Scale in immediate feedback survey and subset into high- and highest-risk groups. Note: N/A

**Figure 5:**
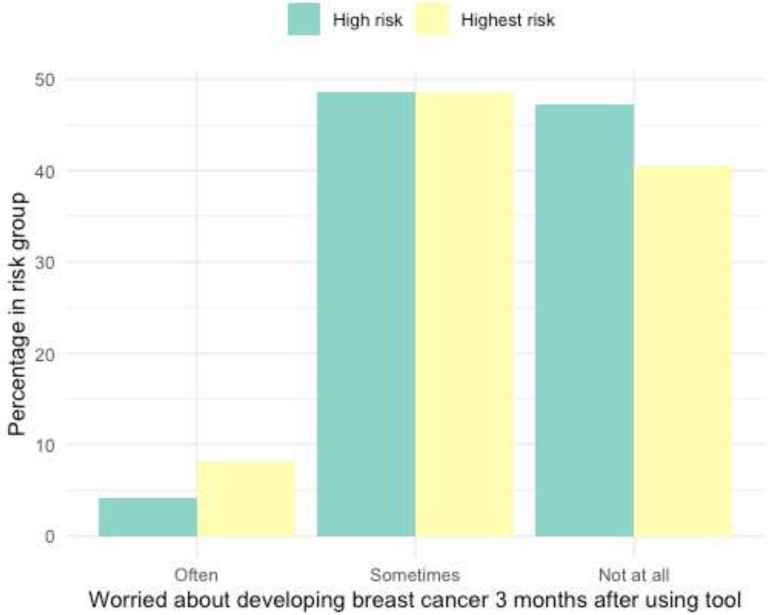
Worry about Developing Breast Cancer (3 month follow up) Bar graph of frequency of worry about breast cancer risk after use of tool. Responses obtained through Likert Scale in 3-month follow up survey and subset into high- and highest-risk groups. Note: N/A

No studies to date have assessed educational tools’ impact on breast cancer anxiety and worry, which is prevalent especially in women with a family history of breast cancer, baseline anxiety, negative illness perceptions, and genetic testing, and impacts decision-making.^40–45^ Providing women with breast cancer risk estimates has minimal negative effects on anxiety but it is unclear if actionable risk reduction strategies from educational tools like the risk assessment tool can have a positive effect.^43,46,47^ In this preliminary investigation of anxiety and worry about breast cancer risk after use of an educational tool, a majority of women report that the tool alleviated or did not affect their emotional state, with no difference noted between high- and highest-risk women (Fig. 4–5, Supplementary Table 2). These findings suggest that greater knowledge regarding one’s risk is not associated with negative emotions and may even alleviate anxiety. It is also possible that providing next steps in risk reduction, as done in the educational tool, empowers women and positively contributes to their emotional well-being.

### Opportunities

Side effects of medications were listed as one of the important reasons that women chose not to take medication to reduce their breast cancer risk. Fortunately, there are now several studies showing that substantially lower doses of tamoxifen are as effective with few side effects.^48^ In addition, new evidence suggests a lower dose of an AI is likely to be just as effective in lowering serum estradiol.^49^

### Limitations

Our study has several limitations. First, the COVID-19 pandemic began during our data collection process, so results may be confounded by the public health crisis. In particular, the lockdown and closure of gyms and recreational centers during the COVID crisis may have contributed to the difficulties in scheduling healthcare appointments. Second, due to the nature of the study, we cannot draw causal conclusions. Third, our results are limited by the smaller sample size in our follow up survey results, and the response rate was 35% thus raising the possibility of response or attrition bias. Lastly, our study used a pre-post design and did not include a control group. Thus, subsequent attitudes and health behaviors following use of the BHD tool may have been affected by other intervening temporal factors beside the tool itself.

We also note that several factors limit the generalizability of our study. The WISDOM study participants who used the risk-assessment tool may share characteristics not reflective of the general population. Our participants were predominantly white and highly educated with no African Americans in the highest-risk group. Furthermore, we did not include participants who were high risk by virtue of pathogenic genetic variants.

### Future improvements in our approach

There is accumulating evidence that the standard breast cancer risk tools, as well as polygenic risk (PRS), identify women with slower growing hormone positive tumors. This means that our current tools are better at identifying the women most likely to benefit from taking medications to lower their risk. We have increased the diversity of the population of the women in WISDOM so future results should reflect this change. We are working on ways to assess which women are benefiting from endocrine risk reducing therapy.^50^ We have modified the tool to educate women about small doses of tamoxifen and exemestane previously described. We are working more directly with primary care groups to determine how to best share risk assessment information about their patients. We are also working to determine if a virtual prevention program can be set up to support women in the WISDOM trial, as well as primary care physicians. Studies are also underway testing new medications to reduce risk in women at risk for developing hormone positive breast cancer. Finally, we can explore partnerships with devices that measure physical activity and diet to assist women in quantifying their lifestyle changes.

## METHODS AND DATA AVAILABILITY

### Modifications of the Risk Assessment Tool

Previously, our team published results of the risk-assessment tool’s pilot study with 17 participants.^33^ We modified the risk-assessment tool based on participants”feedback and updated the references before implementing it to a broader WISDOM study population.

### Study sample

The study sample consisted of 318 WISDOM Study participants in the personalized arm with elevated breast cancer risk in the top 2.5% of BCSC score by age without breast cancer mutation genes (BRCA1, BRCA2, TP53, PTEN, STIK11, CDH1, ATM, PALB2, CHECK2). These high- and highest-risk women are recommended annual mammogram and annual mammogram plus annual MRI screening respectively. Women in the high-risk category are individuals with a 5-year risk greater or equal to 6% in women 65 and older or have a biopsy with atypia and 1^st^ degree family history without chemoprevention. Women in the highest-risk category are individuals with 5-year risk greater or equal to 6% in women 40–64 years old or have a history of chest wall radiation before age 35. Participants eligible for the WISDOM study identify as female, are between ages 40 – 74 years, live in the United States, and have not had prior breast cancer diagnoses. Out of the 318 participants, 109 responded to the follow up survey.

### Salesforce platform

Salesforce is an online platform where study coordinators of the WISDOM study can communicate with and perform coordinator tasks for WISDOM participants. The breast health risk assessment tool was provided through the participants’ Salesforce platforms and was accessible after they log into their WISDOM study portal on their own electronic device. The Salesforce platform allowed study coordinators to visualize whether the risk-assessment tool was ever used through a checkbox function.

### Procedure

High- and highest-risk participants were provided the opportunity to go through the risk-assessment tool with their breast health specialist through a virtual consultation. Previously in the WISDOM study, breast health specialists contacted high- and highest-risk participants to talk about their risk and answer questions. The risk-assessment tool provided a visual aid for the specialist during the discussion. High- and highest-risk participant who did not respond or declined the consultation had the option to use the risk-assessment tool independently.

After participants completed the breast health risk assessment tool once, they were provided the immediate feedback survey found in the last page of the tool. Three months after participants completed their immediate feedback survey, the three-month follow up survey populated their WISDOM portal.

### Data collection

Data was collected from February 2019 to April 2022. A total of 333 participants responded to the feedback survey and 109 participants responded to the three-month follow up survey. Two participants had “stop screening” or “start screening at age 50” recommendations and were excluded from the study. Thirteen completed the survey after being designated low risk and were also excluded from the study.

### Data analysis

Study coordinator MC downloaded immediate feedback survey, three-month follow-up survey data and participant demographics information from the Salesforce platform. Study coordinator TW compiled the demographics and survey information into tables and figures and performed statistical analyses using R studio (version 1.0.153). Pearson’s Chi-squared test was calculated to evaluate for differences between high- and highest-risk group categories.

## Figures and Tables

**Table 1:** Baseline Characteristics of Study Participants Age, BMI, race/ethnicity, education of participants. And further subset for high and highest risk participants.

		High Risk N = 221 (%)	Highest Risk N = 97 (%)	Total Participants N = 318 (%)
Age	40–49	64 (29%)	7 (7.2%)	71 (22.3%)
	50–59	72 (32.6%)	34 (35%)	106 (33.3%)
	60–69	53 (24%)	49 (50.5%)	102 (32.1%)
	70–79	32 (14.4%)	7 (7.3%)	39 (12.3%)
BMI	< 18.5	2 (0.9%)	4 (4.1%)	6 (1.9%)
	18.5 – 24.9	120 (54.3%)	59 (60.8%)	179 (56.3%)
	25 – 29.9	58 (26.2%)	18 (18.6%)	76 (23.9%)
	>30	41 (18.6%)	16 (16.5%)	57 (17.9%)
Race/Ethnicity	White	196 (88.7%)	87 (89.7%)	283 (89%)
	Hispanic	5 (2.3%)	1 (1.0%)	6 (1.9%)
	Black or African American	5 (2.3%)	0	5 (1.6%)
	Asian	2 (0.9%)	3 (3.1%)	5 (1.6%)
	Native Hawaiian or Other Pacific Islander	1 (1.3%)	0	1 (0.31%)
	Two or more races	10 (4.5%)	3 (3.1%)	13 (4.1%)
	Some other race	1 (0.5%)	2 (2.1%)	3 (0.94%)
	No response	0	1 (1.0%)	1 (0.31%)
	Prefer not to answer	1 (0.5%%)	0	1 (0.3%)
Education	High school	7 (3.2%)	2 (2.1%)	9 (2.8%)
	College or technical school	41 (18.6%)	23 (23.7%)	64 (20.1%)
	College graduate or more	173 (78.2%)	71 (73.2%)	244 (76.7%)
	No Response	0	1 (1%)	1 (0.4%)

Note: % calculated in risk groups is out of total participants in each risk group

**Table 2:** Use, Considerations, and Three-Month Follow Up of Breast Cancer Risk-Reducing Strategies Risk reducing strategies (endocrine risk reduction, decreasing alcohol, increasing exercise, etc.) that participants are *already doing before using BHD*, and risk reducing strategies that participants are *considering after using BHD*, obtained from immediate feedback survey with N = 318 respondents. And risk reducing strategies that *they pursued three months later*, obtained from three month follow up survey with N=109 respondents.

	High Risk N = 221 (%)	Highest Risk N = 97 (%)	Total N = 318 (%)	Pearson’s Chi Squared Test *(high- vs. highest-risk participants)*
***Already doing risk reducing activities***				
Medication	7 (3.2%)	8 (8.2%)	15 (4.7%)	p = 0.09
Decrease alcohol	74 (33.5%)	49 (50.5%)^≠^	123 (38.7%)	p = 0.006
Increase exercise	133 (60.2%)	66 (68%)	199 (62.6%)	p = 0.84
Lose weight	82 (37.1%)	45 (46.4%)	127 (39.9%)	N/A
Other	14 (6.3%)	12 (12.4%)	26 (8.2%)	N/A
Nothing	52 (23.5%)	13 (13.4%)	65 (20.4%)	N/A
***Considering risk reducing activities (immediately after using tool)***				
Medication	72 (32.6%)	38 (39.2%)	110 (34.6%)	p = 0.31
Decrease alcohol	33 (14.9%)	14 (14.4%)	47 (14.8%)	p = 1
Increase exercise	76 (34.4%)	22 (22.7%)	98 (30.8%)	p = 0.051
Lose weight	65 (29.4%)	17 (17.5%)	82 (25.8%)	N/A
Other	14 (6.3%)	3 (3.1%)	17 (5.3%)	N/A
Nothing	42 (19%)	22 (22.7%)	64 (20.1%)	N/A
	Highest Risk (N = 72)	Highest Risk (N = 37)	Total (N = 109)	
***Risk reducing activities 3 months after using tool***				
Medication	7 (9.7%)	5 (13.5%)	12 (11%)	p = 0.78
Decrease alcohol	26 (36.1%)	16 (43.2%)	42 (38.5%)	p = 0.6
Increase exercise	34 (47.2%)	19 (51.4%)	53 (48.6%)	p = 0.84
Diet	47 (65.3%)	26 (70.3%)	73 (67%)	N/A
***Would like support services (3 months after using tool)***	30 (41.7%)	17 (45.9%)	47 (43.1%)	N/A

Notes:

% calculated is out of total who either considered endocrine risk reduction, or the total who did not consider endocrine risk reduction from feedback survey response

≠ = statistical significance between high- and highest-risk group

High risk = WISDOM screening assignment recommendation *yearly*, highest risk = WISDOM screening assignment *every 6 months (alternating mammography and MRI)*. Only high- and highest-risk participants receive a breast health specialist consult with the BHD tool. The low-risk participants however have access to the tool to look through on their own.

**Table 3:** Healthcare Risk-Reducing Recommendation for High- and High-Risk Women Table including reasons why participant did not discuss risk with provider, and why they did not pursue endocrine risk reduction, alcohol, or exercise.

	High Risk N = 72 (%)	Highest Risk N = 37 (%)	Total N = 109 (%)
***Discussed risk with provider***	50 (69.4%)	30 (81.1%)	80 (73.3%)
***Healthcare provider recommended following to reduce risk***			
**Medication**	11 (15.3%)	8 (21.6%)	19 (17.4%)
**Decrease alcohol**	9 (12.5%)	6 (16.2%)	15 (13.8%)
**Increase exercise**	11 (15.3%)	11 (29.7%)	22 (20.2%)
**Losing weight**	11 (15.3%)	4 (10.8%)	15 (13.8%)
**Other**	8 (11.1%)	4 (10.8%)	12 (11%)
**Nothing at this time**	18 (25%)	8 (21.6%)	26 (23.9%)

Note: Pearson’s Chi-squared test with Yates ‘continuity correction was performed. No statistical significance noted between high- and highest-risk groups

## Data Availability

The datasets used and analyzed during the study are available from the corresponding author on reasonable request.
